# Supplementary material for: Multilevel DFT Response Theory
Source: J Chem Theory Comput. 2026 May 22;22(11):5649–62. doi: 10.1021/acs.jctc.6c00255 (PMC13255251; doi:10.1021/acs.jctc.6c00255)
Supplement: Supplementary file 1 [file ct6c00255_si_001.pdf]

Supporting Information: Multilevel DFT Response Theory

Alberto Barlini,[†] Julien Bloino,[†] Henrik Koch,[‡] and Tommaso Giovannini*,[¶]

†*Scuola Normale Superiore, Piazza dei Cavalieri 7, 56126 Pisa, Italy.*

‡*Department of Chemistry, Norwegian University of Science and Technology, Trondheim, Norway*

¶*Department of Physics, University of Rome Tor Vergata, and INFN, Via della Ricerca Scientifica 1, 00133, Rome, Italy*

E-mail: tommaso.giovannini@uniroma2.it

S1 para-Nitroaniline in 1,4-Dioxane

Table S1: Calculated and experimental[1] PNA in 1,4-dioxane isotropic total dynamic molar polarizabilities (ζ in cm^3/mol). Gas-phase results are also reported.

	Method	$\zeta(-\omega;\omega)$	$\zeta^{\text{exp}}(-\omega;\omega)$
B3LYP	Gas phase	10.56	14.1 ± 0.4
	QM/PCM	12.83	
	QM/EE	10.95 ± 0.03	
	QM/FQ	13.71 ± 0.08	
	MLDFT$_{AB}$/FQ	12.72 ± 0.07	
	MLDFT$^{\text{pol}}_{AB}$/FQ	13.60 ± 0.07	
CAM-B3LYP	Gas phase	9.91	
	QM/PCM	11.80	
	QM/EE	10.39 ± 0.02	
	QM/FQ	12.74 ± 0.06	
	MLDFT$_{AB}$/FQ	11.85 ± 0.06	
	MLDFT$^{\text{pol}}_{AB}$/FQ	12.59 ± 0.06	

Table S2: Calculated and experimental (EFISHG)[2] PNA in 1,4-dioxane second harmonic hyperpolarizability (Eq. 22 of the main text, in esu). Gas-phase results are also reported.

	Method	$\beta_z^B(-2\omega;\omega,\omega)$	$\beta_z^{B,exp}(-2\omega;\omega,\omega)$
B3LYP	Gas phase	26.14	50.7 ± 1.2
	QM/PCM	53.25	
	QM/EE	33.36 ± 0.36	
	QM/FQ	73.75 ± 1.47	
	MLDFT$^{\text{pol}}_{AB}$/FQ	69.95 ± 1.26	
CAM-B3LYP	Gas phase	19.30	
	QM/PCM	39.19	
	QM/EE	28.15 ± 0.38	
	QM/FQ	62.26 ± 1.52	
	MLDFT$^{\text{pol}}_{AB}$/FQ	58.33 ± 1.27	

Table S3: Calculated and experimental (HRS)[3] PNA in 1,4-dioxane second harmonic hyperpolarizability (in esu). Gas-phase results are also reported. β_{HRS} values are computed according to Ref. 4 and are reported in the B convention.

	Method	$\beta_{\mathrm{HRS}}(-2\omega;\omega,\omega)$	$\beta^{\mathrm{exp}}_{\mathrm{HRS}}(-2\omega;\omega,\omega)$
B3LYP	Gas phase	11.37	32.01
	QM/PCM	22.86	
	QM/EE	21.53 ± 0.23	
	QM/FQ	47.13 ± 0.91	
	$\mathrm{MLDFT}^{\mathrm{pol}}_{AB}$/FQ	44.79 ± 0.77	
CAM-B3LYP	Gas phase	8.48	
	QM/PCM	18.13	
	QM/EE	18.26 ± 0.24	
	QM/FQ	40.00 ± 0.94	
	$\mathrm{MLDFT}^{\mathrm{pol}}_{AB}$/FQ	37.51 ± 0.78	

Table S4: Collection of literature computed and experimental values of the static molar isotropic polarizability $\zeta(0;0)$ (cm^3 mol^{-1}) of PNA in gas-phase and in 1,4-dioxane.

Method	Value	Ref.
**Experimental values**		
Experiment (solution)	404 ± 6	1
**Theoretical data (gas phase)**		
DFT/CAM-B3LYP	275.4	5
CC2	231.9	6
CCSD	239.9	6
**Theoretical data (1,4-dioxane)**		
HF/PCM	418.8	7
DFT/CAM-B3LYP/PCM	373.1	5
CC2/MM	414.5	6
CC2/FQ	576.6	6
CC2-in-MLHF$_{AB}$/FQ	547.2	6
CCSD/MM	416.1	6
CCSD/FQ	568.9	6
CCSD-in-MLHF$_{AB}$/FQ	542.5	6

Table S5: Collection of literature computed and experimental values of the dynamic molar isotropic polarizability $\zeta(\omega;\omega)$ (cm^3 mol^{-1}) of PNA in gas phase and 1,4-dioxane.

Method	Value	Ref.
**Experimental values**		
Experiment (solution)	14.1 ± 0.4	1
**Theoretical data (gas phase)**		
DFT/CAM-B3LYP	9.9	5
CC2	11.0	6
CCSD	11.0	6
**Theoretical data (1,4-dioxane)**		
HF/PCM	11.6	7
DFT/CAM-B3LYP/PCM	11.8	5
CC2/MM	11.9	6
CC2/FQ	12.6	6
CC2-in-MLHF$_{AB}$/FQ	12.2	6
CCSD/MM	10.9	6
CCSD/FQ	11.5	6
CCSD-in-MLHF$_{AB}$/FQ	11.2	6

Table S6: Collection of literature computed and experimental values of the dynamic first hyperpolarizability of PNA in gas phase and 1,4-dioxane.

Method	Property	Value	Units	Ref.
**Experimental values**				
EFISHG^{c,e}	β_z	16.3	10^{-30} esu	8
EFISHG^{c,d}	β_z	16.9 ± 0.4	10^{-30} esu	2
HRS^{b,e}	β_{zzz}	21.3	10^{-30} esu	3
EFISHG^{b,e}	β_{zzz}	32.6	10^{-30} esu	8
EFISHG^{b,e}	β_{zzz}	33.8	10^{-30} esu	2
EFISHG^{b,e} (gas phase)	$\beta_{\parallel}$	9.26 ± 0.38	10^{-30} esu	9
EFISHG^{a,e}	$\zeta_{\parallel}^{(3)}$	120 ± 11	10^{-36} C m^4 V^{-3} mol^{-1}	1
EFISHG^{a,e}	$\zeta_{\perp}^{(3)}$	39 ± 4	10^{-36} C m^4 V^{-3} mol^{-1}	1
**Theoretical data (gas phase)**				
HF^{a,d}	β_z	5.53	10^{-30} esu	10
MCSCF^{a,d}	β_z	9.83	10^{-30} esu	10
MP2^{b,e}	β_{xxx}	21.96	10^{-30} esu	11
CC2^{b,e}	β_{xxx}	24.3	10^{-30} esu	11
DFT^{b,e}	$\beta_{\parallel}$	18.38	10^{-30} esu	12
**Theoretical data (1,4-dioxane)**				
HF/SCRF^{a,d}	β_z	9.06	10^{-30} esu	10
MCSCF/SCRF^{a,d}	β_z	15.07	10^{-30} esu	10
MP2^{b,e}	β_{xxx}	26.75	10^{-30} esu	11
CC2^{b,e}	β_{xxx}	29.08	10^{-30} esu	11
DFT/DRF^{b,e}	$\beta_{\parallel}$	23.94 ± 0.23	10^{-30} esu	12
HF/PCM^{a,e}	$\zeta_{\parallel}^{(3)}$	110	10^{-36} C m^4 V^{-3} mol^{-1}	7
HF/PCM^{a,e}	$\zeta_{\perp}^{(3)}$	36	10^{-36} C m^4 V^{-3} mol^{-1}	7

^{a} Reported according to the B convention.[13]
^{b} Reported according to the T convention.[13]
^{c} Reported according to the B* convention.[13]
^{d} Evaluated at a wavelength of 1060 nm.
^{e} Evaluated at a wavelength of 1064 nm.

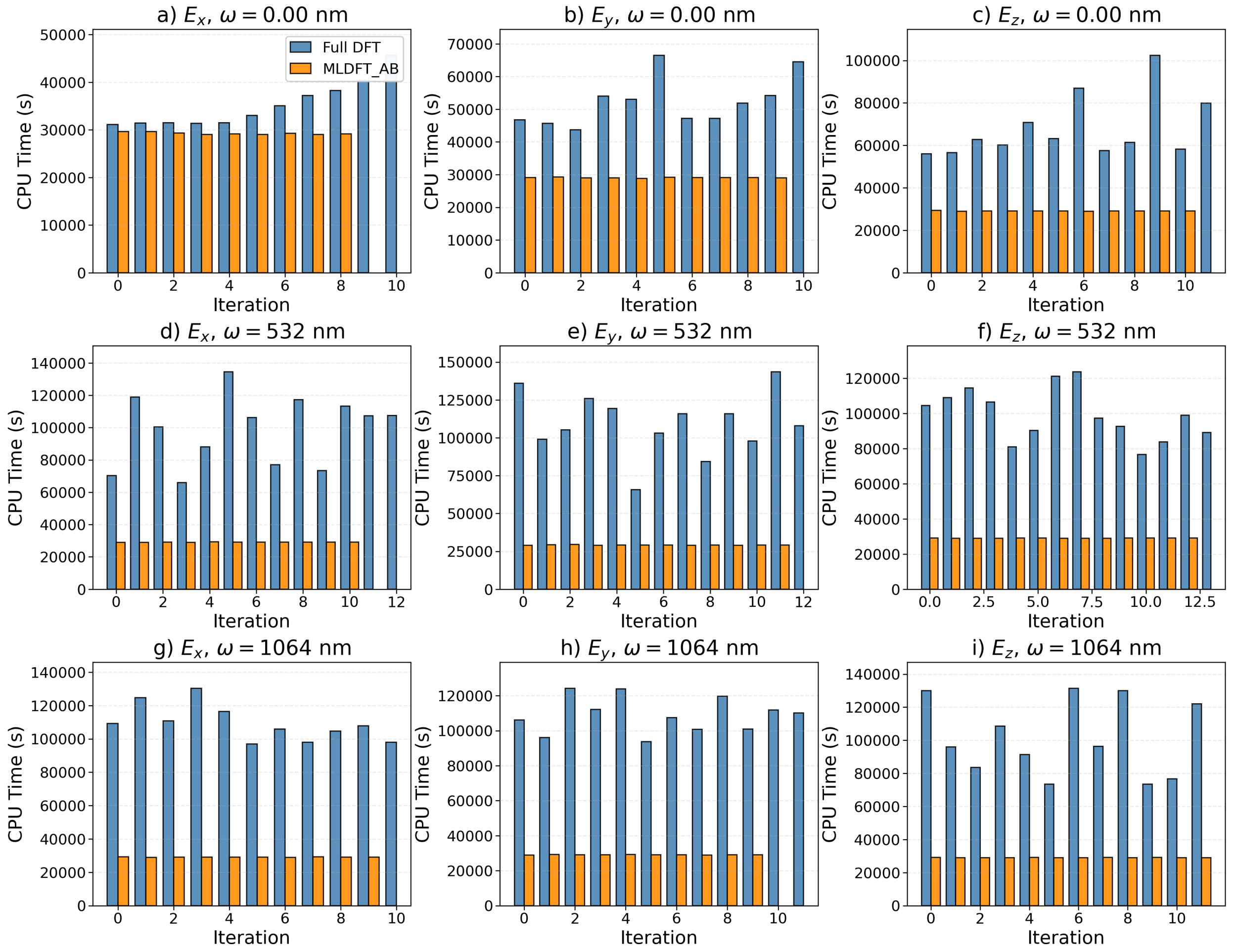

Figure S1: PNA in 1,4-dioxane (snapshot studied in Sec. 4.1 of the main text) full DFT/FQ and MLDFT$^{\text{pol}}_{AB}$/FQ (CAM-B3LYP/aug-cc-pVDZ/6-31G) total CPU times per iteration for each linear response equation.

Table S7: Calculated full DFT/FQ and MLDFT$^{\text{pol}}_{AB}$/FQ (CAM-B3LYP/aug-cc-pVDZ/6-31G) dipole moment (along the z-axis, Debye), static and dynamic isotropic polarizability (cm^3/mol), and static and dynamic ($\lambda = 1064$ nm) parallel components of the first hyperpolarizability with respect to the z-axis (10^{-30} esu) of PNA in 1,4-dioxane.

	full DFT/FQ	MLDFT$^{\text{pol}}_{AB}$/FQ
μ_z	11.73	11.32
$\alpha(0;0)$	44.62	11.18
$\beta_{\parallel}(0;0,0)$	-24.60	-19.46
$\alpha(-\omega;\omega)$	45.28	11.56
$\beta_{\parallel}(-2\omega;\omega,\omega)$	-47.09	-37.42

S2 3-Hydroxybenzoic Acid in Aqueous Solution

Table S8: Calculated and experimental[14] second harmonic hyperpolarizability of HBA in aqueous solution (Eq. 24 of the main text, in esu). Gas-phase results are also reported.

	Method	$\beta_{\mathrm{HRS}}(-2\omega;\omega,\omega)$	$\beta_{\mathrm{HRS}}(-2\omega;\omega,\omega)$
B3LYP	Gas phase	4.16	6.78
	QM/PCM	5.73	
	QM/EE	5.10 ± 0.14	
	QM/FQ	7.97 ± 0.27	
	MLDFT$^{\mathrm{pol}}_{AB}$/FQ	7.58 ± 0.26	
CAM-B3LYP	Gas phase	3.28	
	QM/PCM	7.05	
	QM/EE	4.71 ± 0.12	
	QM/FQ	7.25 ± 0.24	
	MLDFT$^{\mathrm{pol}}_{AB}$/FQ	6.69 ± 0.21	

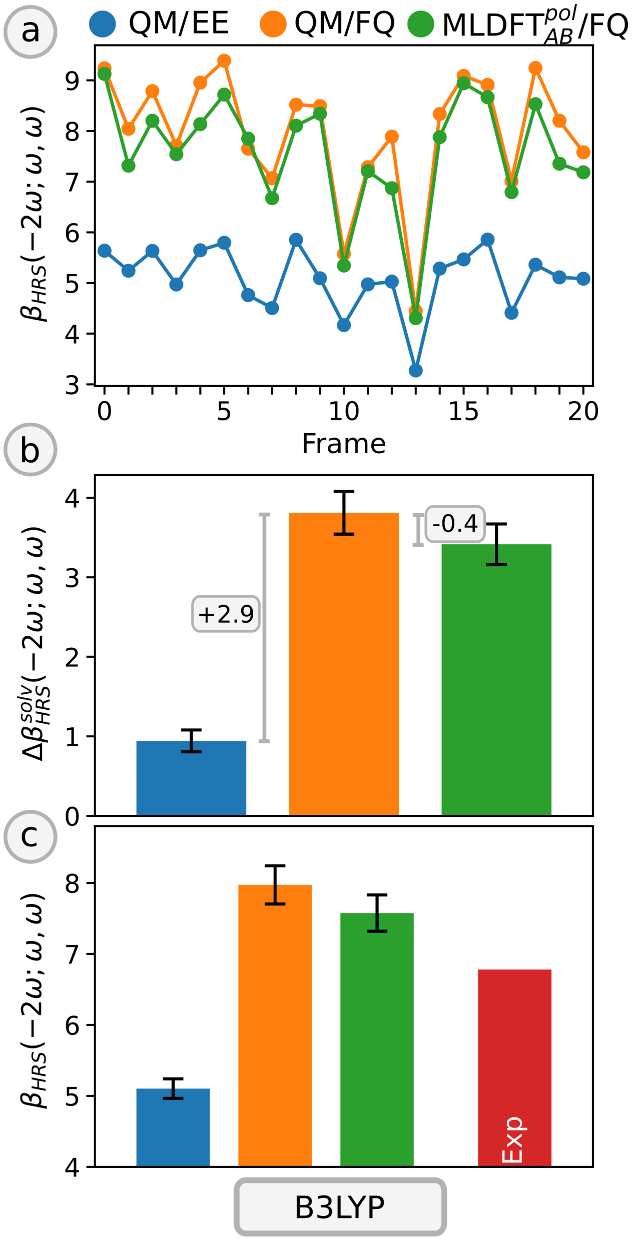

Figure S2: Computed β_{HRS} of HBA in aqueous solution at the B3LYP level. (a) Computed β_{HRS} as a function of the snapshot. (b) Computed solvent effects on β_{HRS} with respect to gas-phase value. (c) Comparison between computed and experimental (from Ref. 14) β_{HRS}. Error bars denote the statistical error. All data are given in esu.

References

(1) Wortmann, R.; Krämer, P.; Glania, C.; Lebus, S.; Detzer, N. Deviations from Kleinman symmetry of the second-order polarizability tensor in molecules with low-lying perpendicular electronic bands. *Chem. Phys.* **1993**, *173*, 99–108.

(2) Teng, C. C.; Garito, A. F. Dispersion of the nonlinear second-order optical susceptibility of organic systems. *Phys. Rev. B* **1983**, *28*, 6766–6773.

(3) Kaatz, P.; Shelton, D. P. Polarized hyper-Rayleigh light scattering measurements of nonlinear optical chromophores. *The Journal of Chemical Physics* **1996**, *105*, 3918–3929.

(4) Plaquet, A.; Guillaume, M.; Champagne, B.; Castet, F.; Ducasse, L.; Pozzo, J.-L.; Rodriguez, V. In silico optimization of merocyanine-spiropyran compounds as second-order nonlinear optical molecular switches. *Phys. Chem. Chem. Phys.* **2008**, *10*, 6223–6232.

(5) Egidi, F.; Giovannini, T.; Piccardo, M.; Bloino, J.; Cappelli, C.; Barone, V. Stereoelectronic, Vibrational, and Environmental Contributions to Polarizabilities of Large Molecular Systems: A Feasible Anharmonic Protocol. *J. Chem. Theory Comput˙* **2014**, *10*, 2456–2464.

(6) Goletto, L.; Gómez, S.; Andersen, J. H.; Koch, H.; Giovannini, T. Linear response properties of solvated systems: a computational study. *Phys. Chem. Chem. Phys.* **2022**, *24*, 27866–27878.

(7) Cammi, R.; Mennucci, B.; Tomasi, J. An attempt to bridge the gap between computation and experiment for nonlinear optical properties: macroscopic susceptibilities in solution. *J. Phys. Chem. A* **2000**, *104*, 4690–4698.

(8) Stähelin, M.; Burland, D.; Rice, J. Solvent dependence of the second order hyperpolarizability in p-nitroaniline. *Chemical Physics Letters* **1992**, *191*, 245–250.

(9) Kaatz, P.; Donley, E. A.; Shelton, D. P. A comparison of molecular hyperpolarizabilities from gas and liquid phase measurements. *The Journal of Chemical Physics* **1998**, *108*, 849–856.

(10) Mikkelsen, K. V.; Luo, Y.; Ågren, H.; Jo/rgensen, P. Solvent induced polarizabilities and hyperpolarizabilities of para-nitroaniline studied by reaction field linear response theory. *The Journal of Chemical Physics* **1994**, *100*, 8240–8250.

(11) Reis, H.; Grzybowski, A.; Papadopoulos, M. G. Computer Simulation of the Linear and Nonlinear Optical Susceptibilities of p-Nitroaniline in Cyclohexane, 1,4-Dioxane, and Tetrahydrofuran in Quadrupolar Approximation. I. Molecular Polarizabilities and Hyperpolarizabilities. *The Journal of Physical Chemistry A* **2005**, *109*, 10106–10120.

(12) Jensen, L.; van Duijnen, P. T. The first hyperpolarizability of p-nitroaniline in 1, 4-dioxane: A quantum mechanical/molecular mechanics study. *J. Chem. Phys.* **2005**, *123*, 074307.

(13) Willetts, A.; Rice, J. E.; Burland, D. M.; Shelton, D. P. Problems in the comparison of theoretical and experimental hyperpolarizabilities. *J. Chem. Phys.* **1992**, *97*, 7590–7599.

(14) Ray, P. C.; Das, P. K.; Ramasesha, S. A comparative study of first hyperpolarizabilities of the acidic and basic forms of weak organic acids in water. *J. Chem. Phys.* **1996**, *105*, 9633–9639.
